# Anti-Remodeling Effects of Xanthohumol-Fortified Beer in Pulmonary Arterial Hypertension Mediated by ERK and AKT Inhibition

**DOI:** 10.3390/nu11030583

**Published:** 2019-03-09

**Authors:** Ana Filipa Silva, Gabriel Faria-Costa, Fábio Sousa-Nunes, Manuel Filipe Santos, Manuel João Ferreira-Pinto, Delfim Duarte, Ilda Rodrigues, João Tiago Guimarães, Adelino Leite-Moreira, Daniel Moreira-Gonçalves, Tiago Henriques-Coelho, Rita Negrão

**Affiliations:** 1 Unidade de Investigação Cardiovascular, Faculdade de Medicina, Universidade do Porto, 4200-319 Porto, Portugal; gabrielfc60@gmail.com (G.F.-C.); fabiosnun@gmail.com (F.S.-N.); geral.m.santos@gmail.com (M.F.S.); manuel.jnfpinto@gmail.com (M.J.F.-P.); amoreira@med.up.pt (A.L.-M.); henriques.coelho@gmail.com (T.H.-C.); 2 Departamento de Cirurgia e Fisiologia, Faculdade de Medicina, Universidade do Porto, 4200-319 Porto, Portugal; danielmgon@fade.up.pt; 3 Departamento de Biomedicina—Unidade de Bioquímica, Faculdade de Medicina, Universidade do Porto, 4200-319 Porto, Portugal; delfimdiogo@gmail.com (D.D.); irodrigues@med.up.pt (I.R.); jtguimar@med.up.pt (J.T.G.); ritabsn@med.up.pt (R.N.); 4 Departamento de Patologia Clínica, Centro Hospitalar Universitário São João, 4200-319 Porto, Portugal; 5 Instituto de Saúde Pública, Universidade do Porto, 4050-600 Porto, Portugal; 6 Centro de Actividade Física Saúde e Lazer, Faculdade de Desporto, Universidade do Porto, 4200-319 Porto, Portugal; 7 Departamento de Ginecologia e Obstetrícia e Pediatria, Faculdade de Medicina, Universidade do Porto, 4200-319 Porto, Portugal; 8 I3S-Instituto de Investigação e Inovação em Saúde, Universidade do Porto, 4200-135 Porto, Portugal

**Keywords:** pulmonary arterial hypertension, polyphenol, remodeling, xanthohumol, beer, monocrotaline

## Abstract

Polyphenols present in some alcoholic beverages have been linked to beneficial effects in preventing cardiovascular diseases. Polyphenols found in beer with anti-proliferative and anti-cancer properties are appealing in the context of the quasi-malignant phenotype of pulmonary arterial hypertension (PAH). Our purpose was to evaluate if the chronic ingestion of a xanthohumol-fortified beer (FB) would be able to modulate the pathophysiology of experimental PAH. Male Wistar rats with monocrotaline (MCT)-induced PAH (60 mg/kg) were allowed to drink either xanthohumol-fortified beer (MCT + FB) or 5.2% ethanol (MCT + SHAM) for a period 4 weeks. At the end of the protocol, cardiopulmonary exercise testing and hemodynamic recordings were performed, followed by sample collection for further analysis. FB intake resulted in a significant attenuation of the pulmonary vascular remodeling in MCT + FB animals. This improvement was paralleled with the downregulation in expression of proteins responsible for proliferation (ERK1/2), cell viability (AKT), and apoptosis (BCL-XL). Moreover, MCT + FB animals presented improved right ventricle (RV) function and remodeling accompanied by VEGFR-2 pathway downregulation. The present study demonstrates that a regular consumption of xanthohumol through FB modulates major remodeling pathways activated in experimental PAH.

## 1. Introduction

Pulmonary arterial hypertension (PAH) is a complex disease characterized by extensive vascular remodeling and vasoconstriction, often culminating in right ventricular (RV) failure [1]. Until very recently, the most common therapies for this disease focused on improving the vasoconstriction/vasodilation imbalance, which were proven to be insufficient, as PAH patient’s prognosis remains dismal [2]. More recently, the extensive vascular remodeling and increased pattern of inflammation have become two major topics of interest in the set of PAH and consequently possible therapeutic targets for this disease.

Epidemiologic studies have demonstrated an association between the consumption of plant-enriched diets and beverages and the improvement in some pathologies like cardiovascular diseases [3]. These benefits have been often related to the presence of polyphenolic compounds and their anti-oxidative properties [4]. Beer is currently one of the most consumed alcoholic beverages in the world [5] and is an important source of polyphenols, which are often indicated responsible for the several described health benefits associated with this beverage consumption [6]. Since beer has its origin mainly from malt and hops, most of the polyphenols found in the beer come from these plants and include several flavonoids, such as chalcones, flavonols, and catechins [7]. Xanthohumol (XN) is a polyphenol almost exclusively present in hops, where it is the principal prenylflavonoid of the female inflorescences. Chemically, it is a prenylated chalcone that may spontaneously be converted into isoxanthohumol during beer production or in the organism, with increased solubility. Isoxanthohumol can then undergo enzymatic *O*-demethylation to 8-prenylnaringenin by intestinal microbiota or by the liver. In recent years, polyphenols, such as xanthohumol, have received more attention, due to other properties that include: (i) they are anti-inflammatory, (ii) anti-proliferative, by interfering with VEGF and MAPK signaling pathways; and (iii) they possess cancer chemo-protective actions, preventing DNA damage, mutation, and uncontrolled cells growth [8,9]. Thus, the production of a xanthohumol-fortified beer has been considered an interesting challenge to the brewing industry.

Given the necessity for a multi-directional therapeutic strategy for PAH, and considering the broad spectrum of actions of polyphenols, it seemed plausible that a polyphenol-enriched alcoholic beverage could have a role in the modulation of PAH. In this work, we aimed to determine whether chronic ingestion of xanthohumol-fortified beer modulates the pathophysiology of experimental PAH.

## 2. Materials and Methods

### 2.1. Reagents

The beer used in this study was regular Superbock^®^ (UNICER Bebidas SA, Porto, Portugal) fortified with Xantho-Flav-Pure (HOPSTEINER, Mainburg, Germany) up to a final concentration of 10 mg/L (FB). To ensure batch-to-batch consistency in polyphenol levels, beer from the same batch was used throughout the whole experiment, assuring the required consistency.

### 2.2. Experimental Design

Animal experiments were performed in accordance to Portuguese law (DL 113/2013), the Declaration of Helsinki, and the European Community guidelines (86/609/EEC) on animal welfare; they also conform to the Guide for the Care and Use of Laboratory Animals published by the US National Institutes of Health (NIH Publication No. 85-23, Revised in 2011). The animal experimental protocol was reviewed and approved by the local committee for animal welfare (Reference number 33/2912/2014).

**Figure 1:**
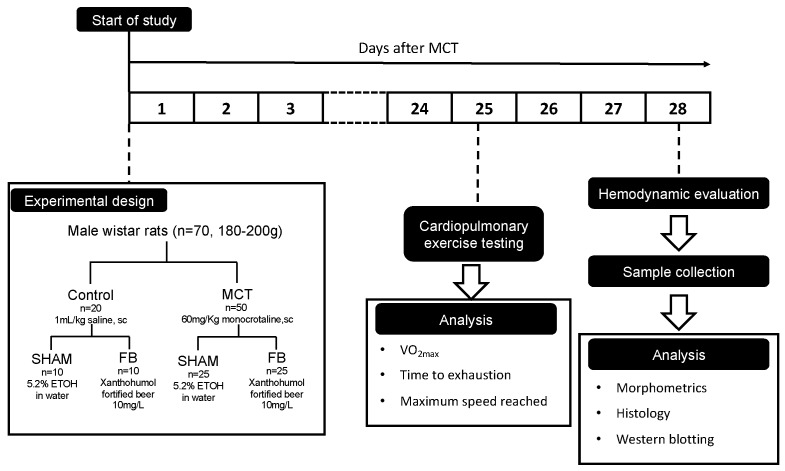
Flow-chart of the experimental design. MCT: monocrotaline, ETOH: ethanol, FB: Xanthohumol-fortified beer.

Male Wistar rats (*n* = 70; weight = 180–200 g; Charles River Laboratories, Barcelona, Spain) were housed in groups of 2 rats/cage and maintained under standard temperature and light conditions (20–22 °C, 12 h light/dark cycle) (Figure 1).

The animals were divided into two major groups: subcutaneously injected with monocrotaline (MCT, 60 mg/Kg, Sigma, Barcelona, Spain), to induce experimental PAH, or with an equal volume of vehicle (Control, 1mL/Kg of saline solution). Immediately after injections, each group was subdivided into another two groups accordingly to the access to the following beverages: 5.2% ethanol solution in water (Control + SHAM, *n* = 10 and MCT + SHAM, *n* = 25) or xanthohumol-fortified beer (Control + FB, *n* = 10 and MCT + FB *n* = 25). All animals were maintained on ad libitum beverages and standard rodent chow. Beverages and animal food were renewed every 2–3 days, with registration of intake as well as the animal’s body weight.

To perform survival analysis, the animals were monitored throughout the protocol and casualties were registered until the last day of experiments (28 days after MCT/saline injection, *n* = 10 for both Control groups, *n* = 15 for MCT + SHAM and *n* = 20 for MCT + FB).

### 2.3. Cardiopulmonary Exercise Testing

Twenty-five days after MCT/saline injection, the rats were submitted to a cardiopulmonary exercise test using motorized treadmill coupled with a gas analyzer (Panlab, Harvard Bioscience Company, Holliston, MA, USA), in which VO2 and VCO2 were continuously recorded. To measure VO2max, each rat performed a 5 min warm-up at 25 cm/s and 10% inclination, followed by treadmill speed increments of 3 cm/s every 2 min until physical exhaustion occurred. Exhaustion was established when the animals accepted three consecutive electric stimuli as opposed to running. VO2max was calculated as an allometric score (mL/Kg^0.75^/min), which is the VO2max/lean body mass ratio.

### 2.4. Hemodynamic Evaluation

Invasive hemodynamic evaluation was performed 28 days after MCT/saline administration using pressure–volume conductance catheters, placed in the right and left ventricles (PVR-1045 and PVR-1035, respectively; Millar Instruments, Houston, TX, USA). Briefly, animals were anesthetized by inhalation of a mixture of sevoflurane and oxygen (8% for induction and 2–3% for maintenance), endotracheally intubated for mechanical ventilation (Dual Mode, Kent Scientific, Torrington, CT, USA), and placed over a heating pad. Under binocular surgical microscopy, the right jugular vein was cannulated for fluid administration (prewarmed 0.9% NaCl solution, 32 mL/Kg/h) to compensate for preoperative losses. After exposing the heart and placing the catheters in the respective ventricles, the animal preparation was allowed to stabilize for 15 min. Hemodynamic recordings were made under basal conditions, with respiration suspended at end-expiration. Data was continuously acquired (MPVS 300, Millar Instruments, Houston, TX, USA), digitally recorded at 1000 Hz (ML880 Powerlab 16/30, Millar Instruments, Houston, TX, USA) and analyzed using Labchart software (AdInstruments, Colorado Springs, CO, USA ). Parallel conductance values were obtained by injection of 10% NaCl bolus through the venous catheter inserted in jugular vein. RV and LV peak systolic pressure (P_max_), end-diastolic pressure (EDP), peak rate for pressure rise and fall (dP/dt_max_ and dP/dt_min_, respectively), constant time of isovolumetric pressure decline (Tau), ejection fraction (EF), and maximal elastance (Ea) were obtained.

### 2.5. Sample Collection

At the end of the hemodynamic evaluation, the animals were euthanized by exsanguination while still under anesthesia. Blood was collected from the RV, centrifuged (5000 rpm, 15 min, 4 °C) in order to obtain plasma and serum sample, and stored at −80 °C until further analysis. Cardiac and lung tissue samples were isolated, weighted, and fixed in 4% paraformaldehyde for microscopy analysis or immediately frozen in liquid nitrogen for molecular biology. Gastrocnemius muscle weight and tibia length were obtained for normalization purposes.

### 2.6. Histology

After fixation, samples from RV and lungs were processed and included in paraffin blocks. Serial sections (4 µm of thickness) were cut using a microtome and mounted on silane-coated slides. Hematoxilin-eosin (HE) and Picrosirius Red staining’s were used in order to determine RV hypertrophy and fibrosis levels, respectively. Sections obtained from lung tissue were also stained with HE to measure pulmonary artery hypertrophy. Six images of random microscopic fields (magnification of × 400) were obtained from each section. Round to ovoid muscle fibers with a nuclear profile were used to measure the RV cardiomyocytes cross-sectional area (CSA; *n* = 800 cardiomyocytes/group). The percentage of cardiac fibrosis was determined dividing the area of fibrotic tissue by the total tissue area. On pulmonary arteries (diameter < 50 μm for small caliber arteries and >50 μm for large caliber arteries, 12/18 arteries per lung per animal), orthogonal intercepts were used to generate eight random measurements of external diameter (distance between the external lamina) and sixteen random measurements of medial thickness (distance between the internal and external lamina). For each artery, medial hypertrophy was expressed as follows: % wall thickness = [(medial thickness × 2)/(external diameter)] × 100. CSA and medial hypertrophy were measured using the Cell B Olympus Software, and fibrosis was obtained using Image J.

### 2.7. Western Blotting

Lung and RV samples (*n* = 8 animals/group) were homogenized with a tissue ruptor (Quiagen, Hilden, Germany) in RIPA lysis buffer (0.5M Tris-HCL, pH 7.4, 1.5M NaCl, 2.5% deoxycholic acid, 10% NP-40, 10 mM EDTA, Millipore, Darmstadt, Germany) supplemented with proteases (P8340, Sigma Aldrich, USA) and phosphatases inhibitors (P5726 and P0044, Sigma Aldrich, St. Louis, MO, USA). Total protein concentration was spectrophotometrically determined with the PierceTM BCA protein assay kit (ThermoFisher Scientific, Waltham, MA, USA) accordingly with manufacture instructions. Simultaneously, a calibration curve was obtained using different concentrations of bovine serum albumin (BSA). Equivalent amounts of total protein (50 µg) were electrophoresed on a 10–12.5% SDS-PAGE at 160 V as described by Laemmli [10]. Gels containing total proteins were transferred to a nitrocellulose membrane (0.45 µm, Whatman^®^, Protran^®^, Sigma Aldrich, St. Louis, MO, USA) in transfer buffer (25 mM Tris,192 mM Glycine, pH = 8.3 and 20% methanol) at 200 mA. Equal protein loading was confirmed by staining membranes with Ponceau S and nonspecific binding was blocked with 5% (w/v) nonfat dry milk or BSA in tris buffered saline with tween-20 (TBS-T, 100 mM Tris, 1.5 mM NaCl, pH = 8.0 and 0.5% Tween 20). Membranes were incubated overnight at 4 °C, with primary antibody solution diluted 1:1000 in 5% (w/v) nonfat dry milk or BSA in TBS-T (rabbit anti-p44/42 MAPK (ERK1/2), cell signaling #9102, rabbit anti-phospho-p44/42 MAPK (ERK1/2), cell signaling #9101; rabbit anti-AKT, cell signaling #9272; mouse anti-phospho-AKT (Ser473), cell signaling #4051; rabbit VEGF receptor (VEGFR)-2, cell signaling #9698; rabbit Caspase-3, cell signaling #9665, rabbit BCL-XL, cell signaling #2762, and rabbit BAX, cell signaling #2772). Afterwards, membranes were washed with TBS-T and incubated with anti-rabbit or anti-mouse secondary antibody (diluted 1:10,000, LI-COR IRDye^®^ 800CW, Lincoln, NE, USA). Immunoreactive bands were observed under fluorescence using an Odyssey system (LI-COR Odyssey, Lincoln, NE, USA) and the results were analyzed with Image StudioTM Lite software v.5.2.5 (LI-COR, Lincoln, NE, USA).

### 2.8. Statistical Analysis

Data analysis was performed using GraphPad V6 software (GraphPad software, San Diego, CA, USA). All data are presented as mean ± SEM and were compared using two-way ANOVA. Normality was tested with a Kolmogorov–Smirnov test and, when verified, a Tuckey post-hoc test was performed to pairwise multiple comparisons. For survival analysis, a Kaplan–Meier survival analysis and a Gehan–Breslow test were performed. Results were considered significantly different when *p* < 0.05.

## 3. Results

### 3.1. FB Effects on Morphometrics

Morphometric data are presented in Table 1.

Both MCT groups presented a pattern of weight loss, as shown by the 15–18% decrease in the final body weight of MCT rats treated with SHAM or FB (*p* < 0.05 vs. respective control groups). This decrease in body weight was accompanied by a decrease in gastrocnemius muscle mass (–19%, *p* < 0.0001 vs. respective control group) and gastrocnemius muscle weight to tibia length ratio (–15%, *p* < 0.01 vs. respective control group). Despite no differences noted in the heart weight to tibia length ratio, MCT + SHAM animals presented RV hypertrophy, as demonstrated by an increase in RV weight (43%, *p* < 0.01 vs. Control + SHAM group), RV weight to tibia length ratio (40%, *p* < 0.01 vs. Control + SHAM group) and RV weight to LV + septum weight ratio (65%, *p* < 0.0001 vs. Control + SHAM group). MCT + SHAM animals also presented an increase in the lungs weight (43%, *p* < 0.0001 vs. Control + SHAM group) and in the lungs weight to tibia length ratio (33%, *p* < 0.01 vs. Control + SHAM group).

FB ingestion in PAH group prevented RV hypertrophy, as observed by an attenuation in RV weight to tibia length ratio (–14%, *p* < 0.05 vs. MCT + SHAM group) and RV weight to LV + Septum weight ratio (–21%, *p* < 0.01 vs. MCT + SHAM group). MCT + FB animals also presented prevention of pulmonary alterations by showing a reduction in lung mass (–15%, *p* < 0.05 vs. MCT + SHAM group) and in lung weight to tibia length ratio (–18%). No significant differences were observed in body weight and gastrocnemius muscle weight in the MCT + FB animals when compared with the MCT + SHAM group. Lastly, alcohol or beer fortified with xanthohumol did not induce significant hepatic or renal toxicity (Table S1).

### 3.2. FB Effect on VO_2max_ and Physical Capacity

**Figure 2:**
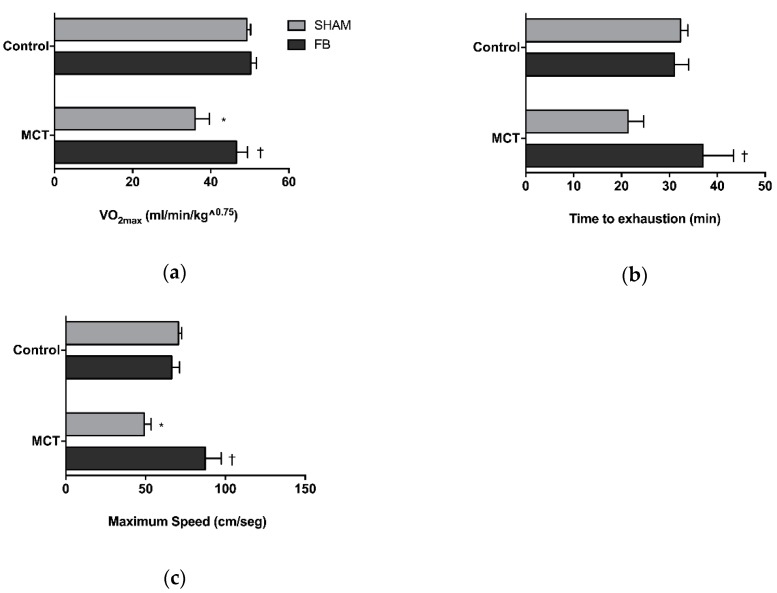
Xanthohumol-fortified beer (FB) ingestion effect on animal VO2max (**a**) time to exhaustion (**b**) and maximum speed that the animals reached on the test (**c**). Data are mean ± SEM of 6 animals per group. * *p* < 0.05 vs. respective control group, † *p* < 0.05 vs. MCT + SHAM. MCT: monocrotaline.

The results of the cardiopulmonary exercise testing are illustrated in Figure 2.

MCT + SHAM animals presented a reduction in the VO2max (−27%, *p* < 0.01 vs. Control + SHAM group) and a diminished maximum running speed (−30%, *p* < 0.001 vs. Control + SHAM group). Animals also presented a non-statistical significant 34% reduction in time to exhaustion. FB intake by PAH rats was able to prevent VO2max decay (*p* < 0.05 vs. MCT + SHAM) and improved physical capacity, as observed by the increase in maximum speed (78%, *p* < 0.001 vs. MCT + SHAM group) and time to exhaustion parameters (73%, *p* < 0.05 vs. MCT + SHAM group). No significant differences were observed between the MCT + FB animals and both healthy control groups. 

### 3.3. FB effect on Pulmonary Remodeling

**Figure 3:**
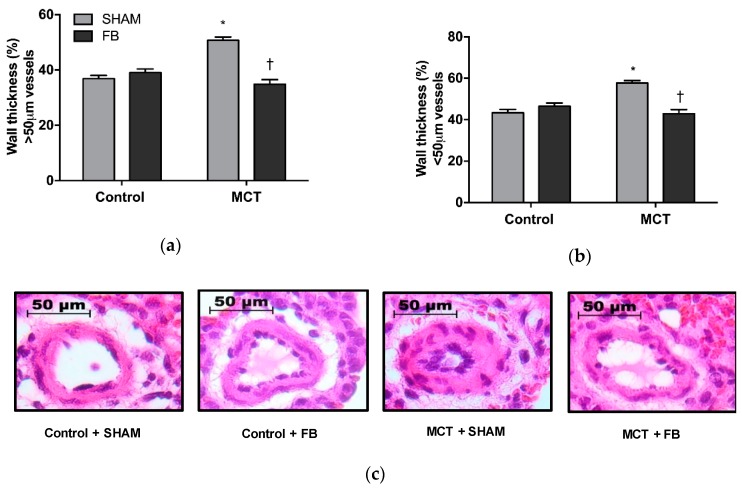
Xanthohumol-fortified beer (FB) intake improved PAH animals pulmonary vascular remodeling. Microscopic analysis of the large (**a**) and small (**b**) intrapulmonary arteries. Representative images of arteries from the different experimental groups (**c**). Data are mean ± SEM from samples of 8 animals per group. * *p* < 0.05 vs. respective control group, † *p* < 0.05 vs. MCT + SHAM. MCT: monocrotaline.

Pulmonary remodeling was observed in the MCT + SHAM animals, as suggested by the increase in the wall thickness of large intrapulmonary arteries (38%, *p* < 0.0001 vs. Control + SHAM group) and small intrapulmonary arteries (33%, *p* < 0.0001 vs. Control + SHAM) (Figure 3a,b, respectively).

MCT + FB animals presented a significant reduction in the wall thickness of both caliber of pulmonary arteries (*p* < 0.0001 vs. MCT + SHAM group), and no differences were detected when compared with the control groups.

#### 3.3.1. ERK1/2 and AKT Expression

**Figure 4:**
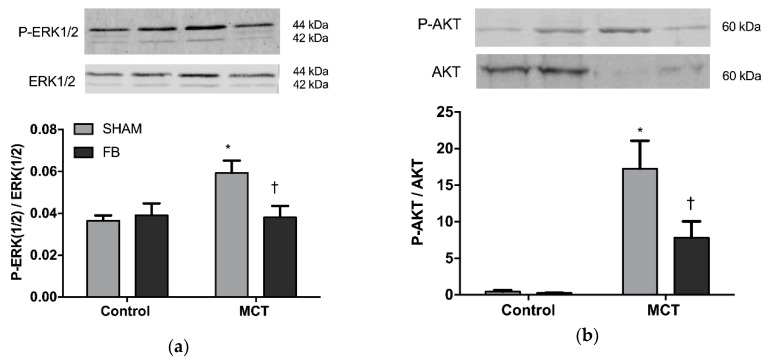
Xanthohumol-fortified beer (FB) effect on pulmonary expression of p-ERK1/2/ERK1/2 ratio (**a**) and p-AKT/AKT ratio (**b**) of healthy (control) or monocrotaline (MCT)-induced PAH animals. Data are mean ± SEM from 8 animals per group. * *p* < 0.05 vs. respective control group, † *p* < 0.05 vs. MCT + SHAM.

In MCT + SHAM animals, the ratio of phosphorylated to total ERK 1/2 was 60% increased in comparison with the Control + SHAM group (*p* < 0.05, Figure 4a). PAH animals that ingested FB displayed a 36% reduction of ERK 1/2 proteins ratio (phosphorylated/total) (*p* < 0.05 vs. MCT + SHAM), returning to values similar to the healthy groups.

AKT phosphorylation was significantly increased in the MCT +SHAM (*p* < 0.001 vs. Control + SHAM) as observed in Figure 4b. This increase in phosphorylation in the PAH group was prevented by FB ingestion demonstrated by a reduction in the ratio phosphorylated/total AKT (*p* < 0.05 vs. MCT + SHAM).

#### 3.3.2. Pulmonary Apoptosis

**Figure 5:**
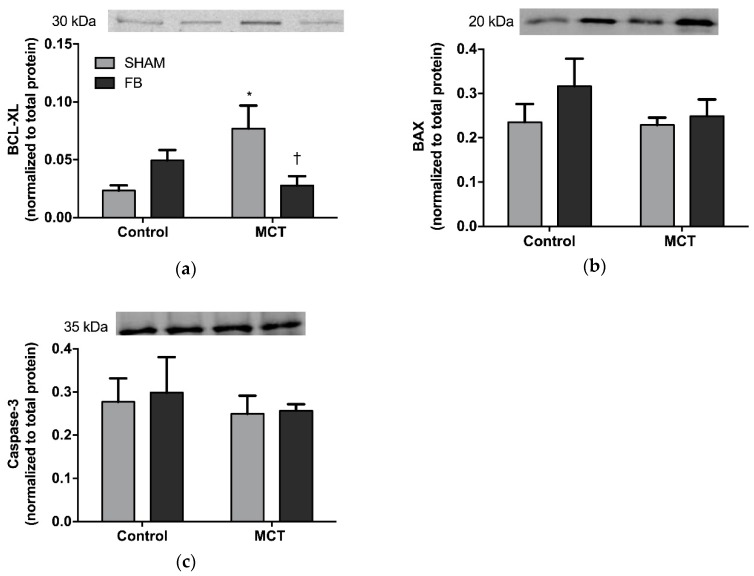
Effect of Xanthohumol-fortified beer (FB) on lung apoptosis of healthy (Control) and monocrotaline (MCT)-induced PAH animals: (**a**) BCL-XL, (**b**) BAX, and (**c**) caspase-3. Data are mean ± SEM from samples of 8 animals per group. * *p* < 0.05 vs. respective control group, † *p* < 0.05 vs. MCT + SHAM.

MCT + SHAM rats presented increased pulmonary expression of the anti-apoptotic protein BCL-XL when compared with the control group (230%, *p* < 0.05 vs. Control + SHAM, Figure 5a).

This elevation was not observed in the MCT + FB group (*p* < 0.05 vs. Control + FB). When analyzing the pro-apoptotic proteins BAX and Caspase-3, no differences were noted in both MCT groups, neither when compared to each other nor when compared to their respective control groups (Figure 5b,c).

### 3.4. FB Effect on RV Hemodynamics and Hypertrophic Phenotype

RV hemodynamic data are summarized in Table 2.

MCT + SHAM animals presented an increase in RV P_max_ (128%, *p* < 0.0001 vs. Control + SHAM group) that was significantly reduced in the MCT + FB group (−20%, *p* < 0.05 vs. MCT + SHAM group). MCT + SHAM group also presented increased RV dP/dt_max_ (74%, *p* < 0.01 vs. Control + SHAM), dP/dt_min_ (123%, *p* < 0.001 vs. Control + SHAM), and EDP (119%, *p* < 0.05 vs. Control + SHAM), together with a decrease in the EF (−46% *p* < 0.01 vs. Control + SHAM).

**Figure 6:**
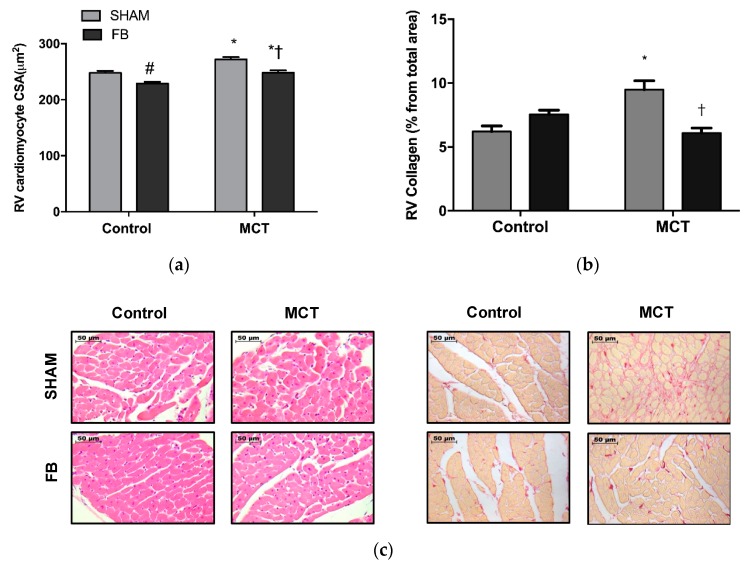

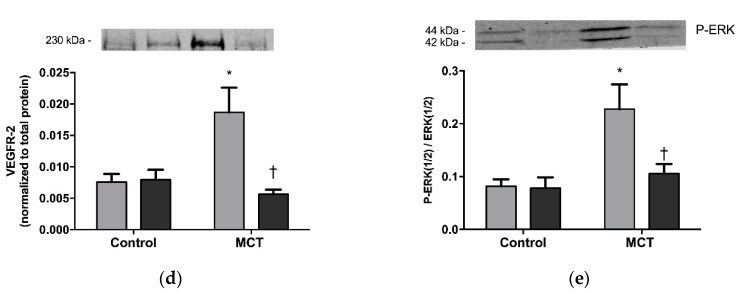
Xanthohumol-fortified beer (FB) improved monocrotaline (MCT)-induced RV remodeling: (**a**) cardiomyocyte cross sectional area, (**b**) fibrosis (demonstrated by the % of collagen deposition), (**d**) VEGFR-2, and (**e**) p-ERK1/2/ERK1/2 ratio. Representative images from CSA and fibrosis from different experimental groups (**c**). Data are mean ± SEM from samples of eight animals per group. * *p* < 0.05 vs. respective control group, # *p* < 0.05 vs. Control + SHAM, † *p* < 0.05 vs. MCT + SHAM. RV: right ventricle, CSA: cross sectional area.

MCT + SHAM showed an increase in the cardiomyocytes cross-sectional area (10%, *p* < 0.0001 vs. Control + SHAM) (Figure 6a,c), that was attenuated in the MCT + FB animals (−9%, *p* < 0.0001 vs. MCT + SHAM group).

MCT + SHAM animals presented higher VEGFR-2 levels in RV (147%, *p* < 0.05 vs. Control + SHAM) as well as increased phosphorylation of the ERK1/2 (102%, *p* < 0.05 vs. Control + SHAM). FB ingestion by MCT animals resulted in a significant reduction of VEGFR-2 expression (−69%, *p* < 0.01 vs. MCT + ETOH) and a non-significantly reduction of p-ERK levels (−23%) (Figure 6d,e, respectively). Regarding cardiac fibrosis, MCT + SHAM animals presented a 44% increase in the RV total collagen deposition (*p* < 0.0001 vs. Control + SHAM) (Figure 6b,c). FB ingestion prevented the excessive collagen deposition and normalized fibrosis levels (*p* < 0.001 vs. MCT + SHAM group).

### 3.5. FB effects on Survival

**Figure 7:**
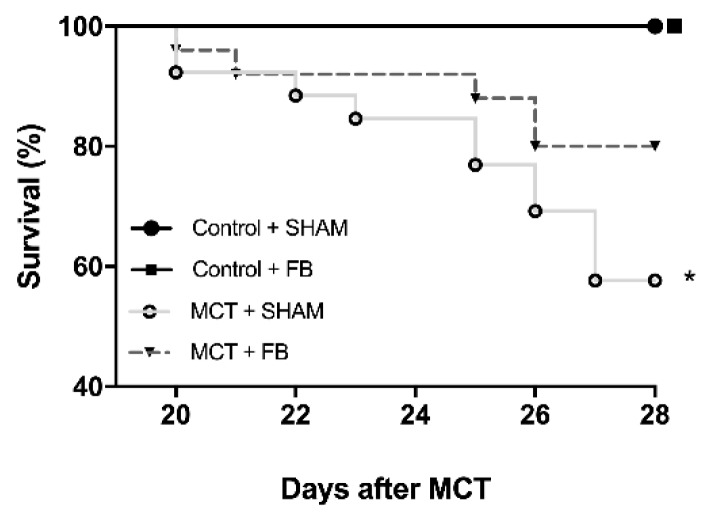
Effect of xanthohumol-fortified beer (FB) on animal survival. The absence of survival curve in Control + SHAM means that all the animals survived until the end of the protocol; the curve is superimposed with Control + FB group. Kaplan–Meier survival curves of the experimental groups are represented according to the days after monocrotaline (MCT) injection. * *p* < 0.05 vs. respective control groups.

The initial number of animals included in the study design was 70 (20 in the control groups and 50 in the MCT groups). This initial number of animals was planned according to our previous experience with the experimental model, and to be the minimum necessary to obtain meaningful results. The MCT animal model of PAH is known for its high mortality. In the MCT + SHAM group, the survival rate (Figure 7) at day 28 was reduced to almost 57% (15 of 25 animals survived until the last day of experiments, *p* < 0.05 vs. Control + SHAM). FB intake improved the survival rate to 80% (20 of 25 animals survived until the last day of experiments) in MCT group.

At the end of the protocol, right ventricle hemodynamic recordings were performed. Because this is an invasive procedure that requires anesthesia and thoracotomy for heart exposure in order to insert the pressure-volume catheters for the hemodynamic recordings, there is always a risk for some animals to not tolerate the surgical stress. Thus, despite surviving until the last day of experiments, some animals did not reach to the hemodynamic procedure (with no sample collection being performed) or died during the recordings, which explains the different number of animals in the tables.

## 4. Discussion

In this study, we first demonstrated that the consumption of a polyphenol-fortified beer has beneficial effects in MCT-induced PAH. The main effects of FB ingestion on experimental PAH were on pulmonary remodeling with the prevention of hypertrophy in the intra-pulmonary arteries. This was due to an improvement in the imbalance between proliferation and apoptosis, through modulation of ERK1/2, PI3K/AKT, and BCL-XL pathways. Consequently, PAH animals that ingested FB presented an enhancement of RV function, decreased hypertrophy, and fibrosis, together with an increase in exercise tolerance and survival.

Pulmonary vascular cells in PAH are characterized by a hyper-proliferative and apoptotic-resistant phenotype, similar to neoplastic cells [11]. In cancer models, this shift has been attributed to deregulated MAPK pathway and its downstream effector ERK1/2 [12,13]. Pulmonary vasculature of animal models and patients with advanced PAH presents this same deregulation, which is associated with a worse outcome [14]. In the present study, we observed increased expression of ERK1/2 as well as its phosphorylated form in the lungs of SHAM animals with PAH. Previous studies from our group reported that xanthohumol (XN) is able to exert direct anti-angiogenic and pro-apoptotic actions on both endothelial and smooth muscle cells, two of the major cell type responsible for the vascular hypertrophy observed in PAH pathogenesis [8]. Herein, we demonstrated an anti-proliferative effect of FB, rich in XN, on the pulmonary vasculature. This effect was mediated by the inhibition of ERK1/2 proteins, a fundamental pathway for cellular proliferation. These results corroborate with previous studies of anti-proliferative effects of XN on several types of neoplastic cells [15], including lung adenocarcinoma [16] mediated by ERK1/2 suppression.

Balance between cell viability and apoptosis is of utmost importance to maintain tissue homeostasis [17]. The PI3K/AKT pathway is crucial for the maintenance of this balance in different cell types [18]. Over-phosphorylation of AKT creates a powerful promotion of cell survival, as it antagonizes and inactivates several components of the apoptotic cascades leading to tumor initiation and progression. In PAH, the PI3K/AKT pathway has been widely recognized as one of the responsible pathways for the abnormalities of vascular smooth muscle cell proliferation and apoptosis [19], highlighting the need to search for pharmacologic inhibitors that could contradict this pattern and potentiate apoptosis [20]. Huang et al. [21] and Pei et al. [22] reported a diminished pulmonary expression of AKT in MCT models, the results of which are consistent with our findings of total AKT. However, in the present study, AKT phosphorylated form increased in MCT + SHAM animals, meaning a higher pulmonary remodeling process. XN has been demonstrated to have anti-angiogenic/pro-apoptotic properties through the inhibition of AKT activation [23,24]. Our results corroborate this observation, since FB ingestion prevented the increase of AKT phosphorylation induced by MCT. Once phosphorylated, AKT is able to confer a resistant phenotype through the inactivation of several factors related to the intrinsic apoptotic cascade, such as BAX, BAD, and caspases-9 and -3, as well as also through the overexpression of anti-apoptotic proteins belonging to the BCL-2 family, such as BCL-2 and BCL-XL [25]. In fact, recent studies on lung cancer cell lines demonstrated that the level of BCL-XL expression could be a key mechanism in controlling the resistance to cell death induced by PI3K/AKT inhibition [26]. In this study, we observed a resistance to apoptosis in the lungs of MCT + SHAM animals, as shown by the increased expression of BCL-XL. Interestingly, this overexpression was significantly attenuated in MCT + FB animals, suggesting that FB can further inhibit the apoptotic resistant phenotype observed in PAH lung vasculature by inhibiting the expression of BCL-XL. Supporting our results, Jiang et al. [27] also found that XN was able to induce apoptosis in in vitro and in vivo cancer models by inhibiting BCL-XL. Nevertheless, FB did not affect the expression of other intrinsic apoptotic proteins such as BAX and caspase-3. Similar results were obtained by Klósek et al. [28] who found that XN alone was too insufficient to cleave caspase-3 or affect the expression of BAX in prostate cancer cells. Delmulle et al. [29] also found in the same type of cancer that XN did not induce any alterations in the expression of caspase-3. Taken together, these results suggest that FB is able to exert pro-apoptotic effects, by interfering with the expression of both PI3K/AKT pathway and the anti-apoptotic protein BCL-XL, which may be an advantage as suggested by Qian et al. They revealed that dual inhibition of PI3K/AKT and BCL-XL had a synergistic effect on apoptosis, proposing that a combined therapy inhibiting these two important pathways would be a major benefit in lung cancer [26].

Ingestion of FB led to an enhancement of RV function and remodeling in MCT group, as suggested by the improvements in RV hemodynamics and hypertrophic phenotype. Yang et al. [30] observed that exposing MCT-induced PAH animals to wine polyphenol resveratrol resulted in the complete normalization of RV dysfunction, through the decrease of parameters such as systolic pressure, free wall thickness, and CSA. Other studies using resveratrol have also found the same pattern of RV normalization [31,32]. However, Wilson et al. [33] reported controversial results about the administration of resveratrol to MCT animals, without significant effects on major RV hypertrophy parameters. Our results corroborate both observations, since despite the improvements in the RV parameters, FB did not provide a complete normalization of the cardiomyocyte CSA. To understand the effects of FB on RV remodeling status of the MCT animals, we analyzed molecular targets directly involved in this process. Several experimental models demonstrated that VEGF is able to induce cardiomyocyte hypertrophy mainly through the activation of VEGFR-2 signaling pathway [34]. Triggering VEGFR-2 was proven to influence the activation of hypertrophic pathways including AKT-1, PKC, and ERK1/2 [35]. In fact, stimulation of cardiomyocytes with phenylephrine resulted in cellular hypertrophy associated with an increase of VEGFR-2 protein levels and a reversion when this receptor gene was silenced [36]. In our study, VEGFR-2 increased in the RV of MCT + SHAM group with an increase in the phosphorylated form of ERK1/2, a VEGFR-2 downstream mediator, corroborating previous results in different animal models [35,37]. To our knowledge, there is no data regarding the direct effects of FB in the cardiomyocyte VEGFR-2 expression and activity. Previous studies of our group demonstrated that XN was able to reverse the increased VEGF levels both in vitro and in vivo studies [8,38]. XN-derived product isoxanthohumol was able to inhibit VEGFR-2 expression in human vascular smooth and endothelial cells and also lead to a decrease in p-ERK1/2 levels [39]. Thus, we can suggest that the RV beneficial effects observed in the MCT + FB can be, at least in part, attributed to polyphenol actions on the VEGF pathway.

Most human [40] and animal data [41] report that PAH subjects present diminished exercise tolerance and VO2max [42], often related to skeletal muscular dysfunction [43]. In our study, MCT + SHAM animals presented a decreased in final body weight with skeletal muscle wasting, both parameters not affected by FB ingestion. Despite that, the MCT + FB group showed an improved VO2max and exercise tolerance that might be related to cardiac function enhancement and improvement of the pulmonary vasculature observed in these animals. Altogether, beneficial effects of FB contributed to the increased survival rate observed.

## 5. Conclusions

Our work demonstrated that FB could prevent the development of experimental PAH. Beneficial effects were observed mainly in the pulmonary vasculature through modulation of pathways centrally involved in PAH pathophysiology such as ERK1/2 and PI3K/AKT. FB also improved RV function and remodeling through a decrease in VEGFR-2 expression. Overall, pulmonary and cardiac improvements provided an increase in physical capacity and animal survival. Despite all the improvements observed in our study, care must be taken when translating the results to the human disease, since rats can differently metabolize the substances studied in this work. In the future, it will be important to understand if FB intake in a late phase of the disease could have similar benefits.

## Figures and Tables

**Table 1:** Xanthohumol-fortified beer (FB) effects on general morphometric characteristics in healthy controls and monocrotaline (MCT) treated animals.

	Control		MCT	
	SHAM (*n* = 10)	FB (*n* = 10)	SHAM (*n* = 12)	FB (*n* = 11)
BW (kg)	0.305 ± 0.0	0.305 ± 0.0	0.250 ± 0.0 **	0.260 ± 0.0 **
HW (g)	0.898 ± 0.0	0.861 ± 0.0	0.879 ± 0.0	0.863 ± 0.0
HW/Tibia (g/mm)	0.027 ± 0.0	0.025 ± 0.0	0.026 ± 0.0	0.025 ± 0.0
RV (g)	0.167 ± 0.0	0.165 ± 0.0	0.239 ± 0.0 *	0.208 ± 0.0
RV/Tibia (g/mm)	0.005 ± 0.0	0.005 ± 0.0	0.007 ± 0.0 *	0.006 ± 0.0 †
RV/LV+S (g/g)	0.309 ± 0.1	0.287 ± 0.0	0.510 ± 0.1 *	0.403 ± 0.0 *†
LV+S (g)	0.586 ± 0.0	0.577 ± 0.0	0.491 ± 0.0 *	0.481 ± 0.0 *
LV+S/Tibia (g/mm)	0.017 ± 0.0	0.017 ± 0.0	0.015 ± 0.0 *	0.014 ± 0.0 *
L (g)	1.323 ± 0.1	1.323 ± 0.1	1.888 ± 0.1*	1.603 ± 0.1 *†
L/Tibia (g/mm)	0.042 ± 0.0	0.035 ± 0.0	0.056 ± 0.0 *	0.046 ± 0.0
Gast (g)	1.921 ± 0.1	2.021 ± 0.1	1.554 ± 0.0 *	1.587 ± 0.0 *
Gast/Tibia (g/mm)	0.055 ± 0.0	0.056 ± 0.0	0.047 ± 0.0 *	0.046 ± 0.0 *

Data are mean ± SEM. * *p* < 0.05 vs. respective control group, ** *p* < 0.001 vs. respective control group, † *p* < 0.05 vs. MCT + SHAM. BW: body weight, HW: heart weight, RV: right ventricle weight, LV+S: left ventricle + septum weight, L: lung weight, Gast: Gastrocnemius weight, SHAM: Animal drinking 5.2% ethanol, FB: fortified beer.

**Table 2:** Xanthohumol-fortified beer (FB) effect on right ventricular hemodynamic evaluation of healthy controls and monocrotaline (MCT)-induced PAH animals.

	Control		MCT	
	SHAM (*n* = 8)	FB (*n* = 8)	SHAM (*n* = 6)	FB (*n* = 9)
P_max_ (mmHg)	27.5 ± 1.5	29.6 ± 1.6	62.7 ± 5.5 *	50.2 ± 3.9 *†
dP/dt_max_ (mmHg/s)	1798 ± 222	1877 ± 190	3133 ± 358 *	2578 ± 104
EF (%)	74.0 ± 1.6	69.9 ± 7.9	39.9 ± 3.6 *	55.2 ± 6.0
EDP(mmHg)	2.1 ± 0.2	2.3 ± 0.9	4.6 ± 0.7 *	3.6 ± 0.4
dP/dt_min_ (mmHg/s)	−1472 ± 84	−1693 ± 174	−3289 ± 528 *	−2671 ± 191
Tau (ms)	8.8 ± 0.8	8.9 ± 1.1	11.5 ± 1.4	9.4 ± 0.7

Data are mean ± SEM. * *p* < 0.05 vs. respective control group, † *p* < 0.05 vs. MCT + SHAM. P_max_: maximum pressure, dP/dt_max_: peak rate of pressure rise, dP/dt_min_: peak rate of pressure fall, EDP: end-diastolic pressure, EF: ejection fraction.

## Supplementary Materials

The following are available online at https://www.mdpi.com/2072-6643/11/3/583/s1, Table S1: Hepatic and renal function in healthy controls and monocrotaline (MCT)-treated animals.

## List of Abbreviations:

ALT	alanine aminotransferase
AST	aspartate aminotransferase
ALP	alkanine phosphatase
BW	Body weight
CSA	Cross-sectional area
dP/dt_max_	Peak rate for pressure rise
dP/dt_min_	Peak rate for pressure fall
Ea	Elastance
EDP	End-diastolic pressure
EDTA	Ethylenediamine tetraacetic acid
EF	Ejection fraction
ERK	Extracellular signal-regulated kinase
FB	Xanthohumol fortified beer
GGT	gamma-glutamyltransferase
Gast	*Gastrocnemius*
HE	Hematoxilin-eosin
HW	Heart weight
L	Lung
LV + S	Left ventricle + septum
MCT	Monocrotaline
PAH	Pulmonary arterial hypertension
P_max_	Peak systolic pressure
RV	Right ventricle
SHAM	Animal drinking 5.2% ethanol
Tau	Constant time of isovolumetric pressure decline
