# Supplementary material for: Anti-Remodeling Effects of Xanthohumol-Fortified Beer in Pulmonary Arterial Hypertension Mediated by ERK and AKT Inhibition
Source: Nutrients. 2019 Mar 9;11(3):583. doi: 10.3390/nu11030583 (PMC6472147; doi:10.3390/nu11030583)
Supplement: Supplementary file 1 [file nutrients-11-00583-s001.pdf]

Supplementary material and methods

Serum biochemical markers were measured in the Central Laboratory, Departament of Clinical Pathology, Centro Hospitalar Universitário São João, using conventional methods with an Olympus AU5400® automated clinical chemistry analyzer (Beckman-Coulter®, Izasa, Porto, Portugal) for the analysis of hepatic and renal toxicity induced by alcohol ingestion. Evaluated parameters included hepatic (aspartate aminotransferase (AST), alanine aminotransferase (ALT) and alkaline phosphatase (ALP), gamma-glutamyltransferase (GGT)) and renal (urea and creatinine) function. A water-drinking control group from our biobank (wistar rats, age-matched, injected with saline solution 1mL/Kg) was added to the analysis as counterfactual to alcohol-drinking groups.

Supplementary Data

Table S1. Hepatic and renal function in healthy controls and monocrotaline (MCT) treated animals.

	**Control + Water**	**Control + SHAM**	**Control + FB**	**MCT + SHAM**	**MCT + FB**
ALT (U/L)	23.75±11.7	29.11±6.0	29.75±6.3	48.5±15.9	35.00±6.2
AST (U/L)	99.50±7.5	99.33±10.4	102.25±23.0	190.60±76.7	98.00±7.5
GGT (U/L)	0.33±0.3	0.71±0.3	0.67±0.7	0.40±0.2	0.40±0.2
ALP (U/L)	95.25±29.8	144.56±21.5	136.33±15.7	151.6±16.3	164.90±13.3
Urea (mg/dL)	43.50±0.3	37.22±2.2	40.00±3.8	36.60±2.0	31.00±3.0
Creatinine (mg/dL)	0.28±0.0	0.25±0.0	0.26±0.0	0.25±0.0	0.19±0.0

Data are mean ± SEM. ALT: alanine aminotransferase, AST: aspartate aminotransferase, GGT: gamma-glutamyltransferase, ALP: alkaline phosphatase.
